# Dual Oncogenic/Anti-Oncogenic Role of PATZ1 in FRTL5 Rat Thyroid Cells Transformed by the *Ha-Ras^V12^* Oncogene

**DOI:** 10.3390/genes10020127

**Published:** 2019-02-09

**Authors:** Michela Vitiello, Giuseppe Palma, Mario Monaco, Anna Maria Bello, Simona Camorani, Paola Francesca, Domenica Rea, Antonio Barbieri, Gennaro Chiappetta, Gabriella De Vita, Laura Cerchia, Claudio Arra, Monica Fedele

**Affiliations:** 1 Institute of Experimental Endocrinology and Oncology “G. Salvatore” (IEOS), National Research Council (CNR), 80131 Naples, Italy; michela.vitiello@gmail.com (M.V.); s.camorani@ieos.cnr.it (S.C.); paolafrancesca1991@libero.it (P.F.); cerchia@unina.it (L.C.); 2 S.S.D. Sperimentazione Animale, Istituto Nazionale Tumori–IRCCS–Fondazione G. Pascale, 80131 Naples, Italy; g.palma@istitutotumori.na.it (G.P.); d.rea@istitutotumori.na.it (D.R.); a.barbieri@istitutotumori.na.it (A.B.); 3 Functional Genomic Unit, Istituto Nazionale Tumori–IRCCS–Fondazione G. Pascale, 80131 Naples, Italy; m.monaco@istitutotumori.na.it (M.M.); a.bello@istitutotumori.na.it (A.M.B.); g.chiappetta@istitutotumori.na.it (G.C.); 4 Department of Molecular Medicine and Medical Biotechnologies, University of Naples “Federico II”, 80131 Naples, Italy; gdevita@unina.it

**Keywords:** Ras oncogene, thyroid cancer, thyrospheres, stem cell biology, FRTL5, PATZ1

## Abstract

PATZ1 is a transcriptional factor downregulated in thyroid cancer whose re-expression in thyroid cancer cells leads to a partial reversion of the malignant phenotype, including the capacity to proliferate, migrate, and undergo epithelial-to-mesenchymal transition. We have recently shown that *PATZ1* is specifically downregulated downstream of the Ras oncogenic signaling through miR-29b, and that restoration of *PATZ1* in Ha-Ras transformed FRTL5 rat thyroid cells is able to inhibit their capacities to proliferate and migrate in vitro. Here, we analyzed the impact of *PATZ1* expression on the in vivo tumorigenesis of these cells. Surprisingly, FRTL5-Ras-PATZ1 cells showed enhanced tumor initiation when engrafted in nude mice, even if their tumor growth rate was reduced compared to that of FRTL5-Ras control cells. To further investigate the cause of the enhanced tumor engraftment of FRTL5-Ras-PATZ1 cells, we analyzed the stem-like potential of these cells through their capacity to grow as thyrospheres. The results showed that restoration of *PATZ1* expression in these cells increases stem cell markers’ expression and self-renewal ability of the thyrospheres while limiting their growth capacity. Therefore, we suggest that PATZ1 may play a role in enhancing the stem cell potential of thyroid cancer cells, but, at the same time, it impairs the proliferation of non-stem cells.

## 1. Introduction

The POZ/BTB and AT-hook-containing Zinc finger protein 1 (*PATZ1*) gene [1], also known as MAZ Related factor *(MAZR)* [2] or Zinc finger Sarcoma Gene *(ZSG)* [3], belongs to the POZ-ZF, also named POK, family of transcription factors which have been implicated in many biological and pathological processes [4,5,6]. In particular, as for other well-known members of this family, such as Bcl-6, PLZF and HIC-1, PATZ1 has been shown to play crucial roles in both development and cancer. Most Patz1^-/-^ mice die perinatally and show embryonic defects, including a general growth retardation, azoospermia, exencephaly, and malposition of the cardiac outflow tract [7,8]. Consistently, *PATZ1* is highly expressed during embryogenesis [2,8] and is still present but at lower levels in all adult tissues [6], where, in some cases, it is expressed exclusively in less differentiated cells [7]. Indeed, PATZ1 is an essential pluripotency regulator of embryonic stem cells since it is integrated in the transcriptional network that regulates the expression of the stem cell key genes *Pou5f1* and *Nanog* [9]. A similar role for PATZ1 has also been suggested in cancer stem cells (CSC) since it is more highly expressed in stem than non-stem cancer derived cells in glioblastomas (GBM) [10]. Despite the fact that a CSC population within a tumor represents a minor subpopulation (~2% of cancer cells), the current idea is that it is responsible for tumor maintenance and progression [11,12]. Indeed, the depletion of the CSC population greatly impairs the tumorigenic potential of the bulk tumor in mouse xenograft models [13,14] and leads to the prolonged survival of tumor-bearing mice [15]. Evidence suggests a role for *PATZ1* in cancer, either as an oncogene, tumor suppressor, or double oncogene/tumor suppressor, depending on the tumor type [6]. In thyroid cancer, *PATZ1* expression has been investigated in human thyroid cancer specimens and found to be downregulated with respect to normal thyroid tissue and increasingly downregulated going from well differentiated papillary carcinomas to poorly differentiated and anaplastic carcinomas, which suggests a tumor suppressor role involved in counteracting thyroid cancer progression toward a less differentiated phenotype [16,17]. This hypothesis has been recently sustained by in vivo studies in *Patz1*-ko mice mated with mice transgenic for the thyroid specific oncogene *RET/PTC1*, which showed that homozygous deletion of the *Patz1* gene worsens the thyroid cancer outcome in RET/PTC1 mice, by inducing the development of anaplastic thyroid carcinomas (ATC) and solid variants of papillary thyroid carcinomas (PTC) [18]. Restoration of *PATZ1* expression in human thyroid cancer cells partially reverts their malignant phenotype [16,17], whereas its silencing induces malignant transformation of normal thyroid cells [17], thus confirming a tumor suppressor role for *PATZ1* in thyroid carcinogenesis.

Downregulation of *PATZ1* in thyroid cancer appears to be a crucial event downstream of the Ras signaling. Indeed, in FRTL5 rat thyroid cells, *PATZ1* expression is specifically downregulated upon transformation with the *Ha-Ras^V12^* oncogene, and re-expression of *PATZ1* causes a partial reversion of the transformed phenotype in terms of proliferation and migration ability [19]. FRTL5-Ras cells represent a valuable in vitro model of thyroid malignant transformation, in which the oncogene *Ha-Ras^V12^* is able to induce an undifferentiated phenotype characterized by a high migratory and invasive aptitude [20,21,22]. However, no studies have so far analyzed the stemness potential of these cells.

Here, we did an in vivo tumorigenic assay by injecting cells subcutaneously in nude mice to analyze the impact of *PATZ1* expression on the capacity of Ras-transformed FRTL5 cells to develop tumors. The unexpected result that tumor engraftment was enhanced in mice injected with *PATZ1*-expressing cells compared to controls led us to evaluate the impact of *PATZ1* on the stem cell potential of these cells. To this aim, in vitro functional assays (sphere-forming capacity and efficiency) and expression analysis of markers of stemness in adherent parental cells and sphere-forming cells were carried out to reveal and quantify the presence and self-renewal capability of stem-like cells in FRTL5-Ras cells with or without overexpressed *PATZ1*.

## 2. Materials and Methods 

### 2.1 FRTL5-Ha-Ras^V12^ Cell Model and its Derivative FRTL5-Ras-PATZ1

FRTL5-Ras cells are rat thyroid FRTL-5 cells stably expressing the human *Ha-Ras^V12^* oncogene (clone V29) [20]. FRTL5-Ras-PATZ1 cells were obtained by transfection of FRTL5-Ras cells with a PATZ1 expression plasmid (clones PA22 and PA28) or backbone vector (clones BV8 and BVMP) (Figure S1), as previously reported [19]. All FRTL-5 cells derivatives have been cultured in Ham’s F12 medium Coon’s modified (Sigma, St. Louis, MO, USA) supplemented with 5% calf serum (Life Technologies, Inc., Carlsbad, CA), penicillin (100 U/mL) (Sigma), and streptomycin (100 mg/L) (Sigma) and in the presence of a mix of six growth factors (10 nM TSH, 10 nM hydrocortisone, 100 nM insulin, 5 mg/mL transferrin, 5 nM somatostatin, and 20 μg/mL glycyl-histidyl-lysine) in a 5% CO_2_ atmosphere.

### 2.2. Tumor Engraftment in Nude Mice

To test in vivo tumorigenicity, 2 x 10^6^ FRTL5-Ras-PATZ1 and FRTL5-Ras-Control (Ctrl) cells were inoculated subcutaneously into six immunodeficient nude (8 weeks old) Foxn1 nu/nu female mice per cell group. The number of animals had been calculated through the Mead’s resource equation to use the minimum number of mice that allow the chance of getting statistically significant differences [23]. Cells, previously tested to be mycoplasma-free, were all collected at the same time, resuspended at a concentration of 2 x 10^6^ in 200 μL PBS, and injected into the left posterior flank of each mouse, previously marked for identification. All injections were done in the same day. The mice were then housed all together until the end of the experiment. One mouse injected with FRTL5-Ras-Ctrl cells was excluded from the analysis because it died during the experiment for unknown reasons. Tumor occurrence was monitored by measuring with calipers at least once every four days. Tumor volume was determined as (length x width^2^)/2. Mice were sacrificed when tumor reached the cutoff of 1500 mm^3^ or at 130 days post injection (end of the experiment for animal aging). All mice were maintained under standardized nonbarrier conditions in the Laboratory Animal Facility of Istituto dei Tumori di Napoli (Naples, Italy). All studies were conducted in accordance with the 3Rs principle and Italian regulations for experimentations on animals (prot. no. 937/2013 approved by the Italian Ministry of Health on 23 September 2013).

### 2.3. Histopathological Analysis and Immunohistochemistry

Histological evaluation was performed on all xenograft tumors which were excised when mice were sacrificed, fixed overnight in 10% neutral buffered formalin, and embedded in paraffin. Sections (5 µm) were stained with hematoxylin and eosin. Blinded (i.e., without knowledge of genotype) histological evaluation included the characterization of tumor morphology and diagnosis. Immunohistochemical staining was performed on 5 µm paraffin sections. Endogenous peroxidase was inhibited by 0.3% hydrogen peroxide in methanol for 30 min. For antigen retrieval, slides were microwaved in a DAKO autostainer (Agilent Technologies, Cernusco sul Naviglio, MI, Italy) in 0.01 M citric acid for 10 min and then quenched in 1% H_2_O_2_.

For immunohistochemistry, the following primary antibodies were used: anti-Ki67 (D3B5, 1:25, Cell Signaling Technology, Danvers, MA, USA); anti-E-cadherin (G-10, 1:50, Santa Cruz biotechnology, Dallas, TX, USA); anti-PATZ1 (R1P1 custom clone [24], Primm, Milan, Italy). The secondary antibody was biotinylated goat anti-rabbit or anti-mouse antibody (Vector Laboratories, Burlingame, CA, USA). Specific binding was amplified using the streptavidin-biotin immunoperoxidase technique (DAKO). The chromogen reaction was developed with 3–3 diaminobenzidine (DAB) solution (DAKO), and nuclei were counterstained with Mayer’s hematoxylin. Negative controls were performed by omitting the primary antibody. At least 10 high-power fields, randomly chosen, have been analyzed for each tumor.

### 2.4. Terminal Deoxynucleotidyl Transferase Mediated Deoxy Uridine Triphosphate Nick End Labeling Assay

The FragEL™ DNA Fragmentation Detection assay kit (CalBiochem, CA, USA), a Terminal Deoxynucleotidyl Transferase Mediated Deoxy Uridine Triphosphate Nick End Labeling Assay (TUNEL) assay, was used to investigate apoptosis induction on paraffin embedded tissue specimens according to the manufacturer’s protocol. Tissue slides were deparaffinizated, rehydrated, and permeabilizated with 20 µg/mL proteinase K at room temperature for 20 min. The inactivation of endogenous peroxidases was performed with 3% H_2_O_2_ at room temperature for 5 min. After washing with equilibration buffer, the labeling reaction was performed by incubating cells with 60 µL of terminal deoxynucleotidyl transferase (TdT) labeling reaction mixture at 37 °C for 1.5 h in the dark. The samples were then incubated with a streptavidin-horseradish peroxidase (HRP) conjugate at room temperature for 30 min. After TBS washing, 100 μL of DAB solution was placed in each slide for 15 min. For the counterstaining of sections, methyl green counterstaining solution was used at room temperature for 3 min. At least 10 high-power fields, randomly chosen, have been analyzed for each tumor.

### 2.5. Spheroid-Forming and Self-Renewal Assays

7000 cells/well were seeded in 6-well plates previously coated with a thin layer of 1% agarose in Ham’s F-12 medium and Coon’s modification supplemented with 20 ng/mL Fibroblast Growth Factor (FGF, Sigma), 10 ng/mL Epidermal Growth Factor (EGF, Sigma), and 2% B27 (GIBCO, ThermoFischer Scientific, Waltham, MA, USA), and incubated at 37 °C in 5% CO_2_. After 14 days of culture, the size and number of spheres (primary thyrospheres) were analyzed under the light microscope. All spheroids with a diameter > 60 μm were counted to calculate sphere forming efficiency (SFE) obtained as follows: Sphere number/cells plated x 100. To calculate differences in size, spheres were sub-divided in small (diameter < 100 μm), medium (diameter > 100 μm; < 200 μm), and large (diameter > 200 μm).

Primary thyrospheres were reduced as single cells both mechanically and by trypsinization. 7000 cells/well were seeded in 6-well plates as described above. After 14 days of culture, size and number of spheres (secondary thyrospheres) were analyzed under the light microscope. All spheroids with a diameter > 60 μm have been counted to calculate self-renewal ability obtained as follows: number of secondary thyrospheres/number of primary thyrosphere cells plated × 100.

### 2.6. RNA Extraction and qRT-PCR

Total RNA was isolated from cells using TRI-reagent solution (Sigma) and treated with DNase (Invitrogen, ThermoFisher Scientific). Reverse transcription was performed using random hexanucleotides as primers and highscript reverse transcriptase (Bio-Rad, Hercules, CA, USA) according to standard procedures. Quantitative (q) PCR was carried out using the power SYBR Green PCR Master Mix (Bio-Rad), according to manufacturer’s instructions. Primers specific for rat *G6pd* were used for normalization of the data. Primer sequences were as follows: rat *Pou5f1* (5′-CCTGCAGCAGATCACTAGCAT-3′ / 5′-CACTCGAACCACATCCCTCT-3′); rat *Sox2* (5′-ACCGTGATGCCGACTAGAAA-3′ / 5′-GCGCCTAACGTACCACTAGAA-3′); rat *Nanog* (5′-GTCTGCTACTGAGATGCTCT-3′ / 5′-ATCTGCTGGAGGCTGAGGTA-3′); rat *G6pd* (5′-TCCTCTATGTGGAGAATGAACG-3′ / 5′-TCATTCAGAGCTTTGCCACA-3′); rat *Patz1* all variants (5′-CCAGAGCTGTGGGAAAGG-3′ / 5′-TGCACCTGCTTGATATGTCC-3′); rat *Cdh1* (5′-CCTGGGACTCCAGTTACAGG-3′ / 5′-CTC.AGACCCTGTGAAAGCTGG-3′); rat *Vim* (5′-GAATACCGGAGACAGGTGCAG-3′ / 5′-CGGCCAATAGTGTCCTGGTAG-3′).

### 2.7. Statistical Analysis

The experiments were performed in independent biological triplicates, and the results are expressed as the mean ± standard error (SE). The one-way ANOVA followed by Tukey’s multiple comparison test was used to compare more than two groups of data. Means of two groups of data were compared by Mann–Whitney or Student’s *t*-test. Tumor engraftment was analyzed comparatively by Kaplan-Meyer curves followed by Log Rank test. A *p*-value < 0.05 was considered to be statistically significant for all statistical tests.

## 3. Results

### 3.1. PATZ1 Expression Enhances Tumor Onset of FRTL5-Ras Mouse Xenograft Models

**Figure 1:**
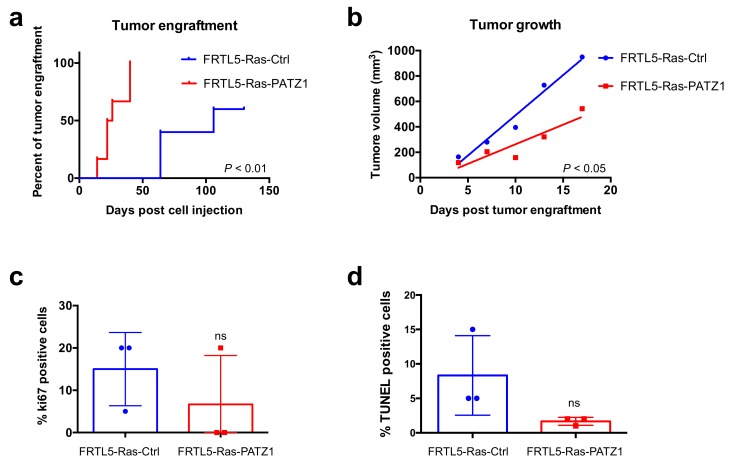
*PATZ1* enhances tumor engraftment of FRTL5-Ras cells but inhibits their growth rate. (**a**) Kaplan-Meier curves analyzing tumor engraftment (first appearance of a palpable mass) in cohorts of 6 or 5 mice/group injected with either FRTL5-Ras-PATZ1 or FRTL5-Ras-Control (Ctrl) cells, respectively. As assessed by Log Rank test, the difference between the curves were highly significant (*P* < 0.01); (**b**) tumor growth in the two cohorts of animals, considering the first 17 days post engraftment, was significantly different (*P* < 0.05), as assessed by the analysis of linear regression (F = 9.58; DFn = 1; DFd = 6). Mean values ± SE have been used to calculate the slopes. (**c**) Percentage of tumor cells stained positively for Ki67 proliferation marker in FRTL5-Ras-Ctrl and FRTL5-Ras-*PATZ1* xenografts. Differences among means were not significant according to the Mann Whitney test (*P* = 0.2); (**d**) percentage of tumor cells positive at TUNEL assay. Differences among means were not significant according to the Mann Whitney test (*P* = 0.1). ns, not significant.

To investigate in vivo the impact of *PATZ1* expression on the transformed phenotype of FRTL5-Ras cells, we analyzed the capacity of Ras-transformed rat thyroid cells stably transfected with a *PATZ1*-expressing plasmid (FRTL5-Ras-PATZ1), compared to control cells transfected with a backbone vector (FRTL5-Ras-Ctrl) (Figure S1), which were previously generated in our laboratory [19], to develop tumors when injected in athymic nude mice. To this aim, two groups of 6 mice/each were injected with either FRTL5-Ras-PATZ1 or FRTL5-Ras-Ctrl cells. Surprisingly, FRTL5-Ras-PATZ1-injected mice developed tumors at least one month earlier than FRTL5-Ras-Ctrl controls, and tumor engraftment rate was 100% (6 out of 6) in mice injected with FRTL5-Ras-PATZ1 cells, against 60% (3 out of 5; one mouse died for unknown reasons and was excluded from the experiment) in mice injected with control cells (Figure 1a). Kaplan–Meyer curves analyzing tumor engraftment in function of the time from injection, followed by Log-rank (Mantell–Cox test), showed a highly significant difference between FRTL5-Ras-PATZ1-injected mice and their controls (*P* = 0.0018; HR = 0.1914; 95% CI 0.01456-0.2701) (Figure 1a). However, the median tumor growth/group, normalized to the first appearance (engraftment) of each tumor and followed for 17 days post engraftment, was significantly different (Figure 1b). Indeed, three FRTL5-Ras-PATZ1 tumors either completely regressed after 24 days (2 out of 6) from injection or did not reach a volume over 150 mm^3^ (1 out of 6) up to the end of the experiment (130 days post injection) (data not shown). Consistently, only 1 of 3 FRTL5-Ras-PATZ1 tumors analyzed, excised at the end of their growth observation, showed Ki67 positivity against 3 out of 3 from control group, even though no significant differences in Ki67 tumor expression—likely due to the low number of specimens analyzed—was detected in the two cohorts of mice (Figure 1c). The apoptotic rate of the tumors derived FRTL5-Ras-PATZ1 cells, analyzed by TUNEL assay, was not increased compared to controls (Figure 1d), thus confirming that the slower growth rate of these tumors was likely due to a proliferative defect. Interestingly, the histopathological analysis of the tumors revealed that—unlike all tumors derived from FRTL5-Ras-Ctrl cells constituted by an anaplastic phenotype with spindle cell morphology resembling mesenchymal cells—in one of the tumors derived from FRTL5-Ras-PATZ1 cells, there were areas in which follicular-like structures, surrounded of epithelioid cells, showing a diffuse cytoplasmic positive immunostaining for E-cadherin, were observed (Figure S2a). The analysis by qRT-PCR of the E-cadherin encoding gene (*Cdh1*) confirmed its enhanced expression in tumor xenografts derived from *PATZ1*-expressing cells compared to controls (Figure S2b). Consistent with previous results in xenografts from *PATZ1* expressing human thyroid cancer cells [16], PATZ1 expression tends to be lost even in tumors derived from *PATZ1* overexpressing cells and is mainly observed in tumor areas characterized by follicular-like structures (Figure S2a).

### 3.2. PATZ1 Expression Enhances Self-Renewal Ability of Thyroid Cancer Stem-Like Cells

To investigate the cause of the enhanced tumor engraftment of FRTL5-Ras-PATZ1 cells we evaluated whether *PATZ1* expression plays a role in thyroid cancer stem biology. Accordingly, we analyzed the growing capacity as spheroids of FRTL5-Ras-PATZ1 compared to FRTL5-Ras-Ctrl cells. The capacity to grow in vitro as spheroids in ultra-low adherence attachment conditions and opportune medium is indeed an exclusive characteristic of stem and cancer stem-like cells [25].

**Figure 2:**
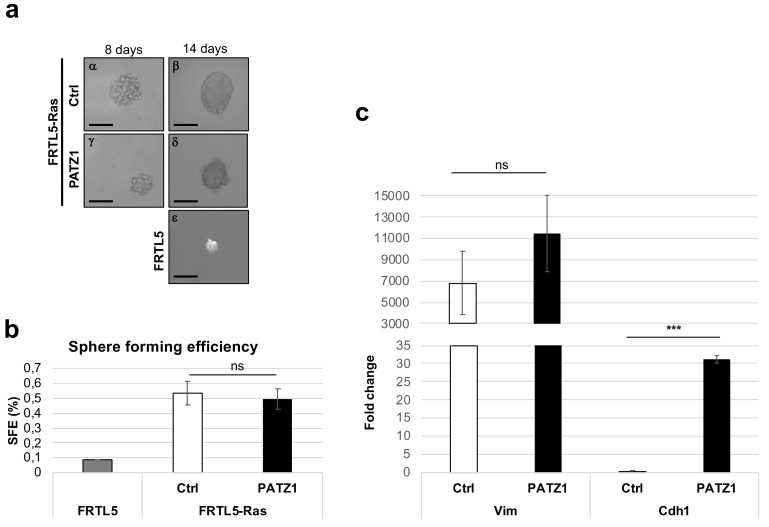
Thyrosphere formation in rat thyroid cells. (**a**) Representative images of P0 thyrospheres obtained from FRTL5-Ras-Ctrl, FRTL-Ras-PATZ1, and FRTL5 cells, at 8 and 14 days post plating in stem cell medium. Scale bars: 100 μm; (**b**) sphere forming efficiency calculated at 14 days post plating cells in stem cell medium. No significant differences were observed between FRTL5-Ras-*PATZ1* and FRTL5-Ras-Ctrl cells. The graph expresses the mean values of three independent experiments performed in two different clones for either Ctrl or *PATZ1* cells. FRTL5 untransformed cells were used as negative control in one experiment performed in duplicate. (**c**) qRT-PCR analysis of Vimentin (*Vim*) and E-cadherin (*Cdh1*) gene expression in thyrospheres from FRTL5-Ras-Ctrl and FRTL-Ras-PATZ1 cells. The data shown express the mean values ± standard error (SE) of three independent experiments performed in triplicate, and, relative to the levels of *Cdh1* expression in control thyrospheres. ns, not significant; ***, *P* < 0.001 as assessed by unpaired *t*-test.

Therefore, two FRTL5-Ras-PATZ1 cell clones and two controls were grown in ultra-low adherence conditions which was obtained by stratifying a thin agarose layer on the culture dishes in a stem cell medium containing FGF, EGF, and B27 in absence of serum. Starting from 8 days from plating, either FRTL5-Ras-Ctrl or FRTL-Ras-PATZ1 cells formed well-structured spheroids, here named thyrospheres as previously described [26], consistent with the presence of a cancer stem-like cell subpopulation (Figure 2a, left panels). At 14 days, when cells appear fused together and individual cells cannot be identified (Figure 2a, right panels), all thyrospheres (primary thyrospheres P0) were counted and measured under the light microscope. The sphere-forming efficiency, calculated as the percentage of thyrospheres formed with respect to the number of cells plated, was not different between the two experimental groups (0.54% ± 0.08 and 0.50 ± 0.07, for FRTL5-Ras-Ctrl and FRTL5-Ras-PATZ1, respectively), suggesting that the number of stem-like cells was not different inside the two cell lines (Figure 2b). Conversely, FRTL5 untransformed cells formed only few small spheres at 14 days post plating (Figure 2a,b). Characterization of epithelial-to-mesenchymal transition (EMT) markers by qRT-PCR revealed that these thyrospheres express high levels of the gene coding for vimentin (*Vim*), without significant differences between the two groups, indicating they were all mesenchymal like, independently from the expression of *PATZ1*. However, a significant increase in the gene coding for E-cadherin (*Cdh1*) was observed in FRTL5-Ras-PATZ1 spheroids (Figure 2c), suggesting a partial mesenchymal-to-epithelial (MET) induction by *PATZ1*.

**Figure 3:**
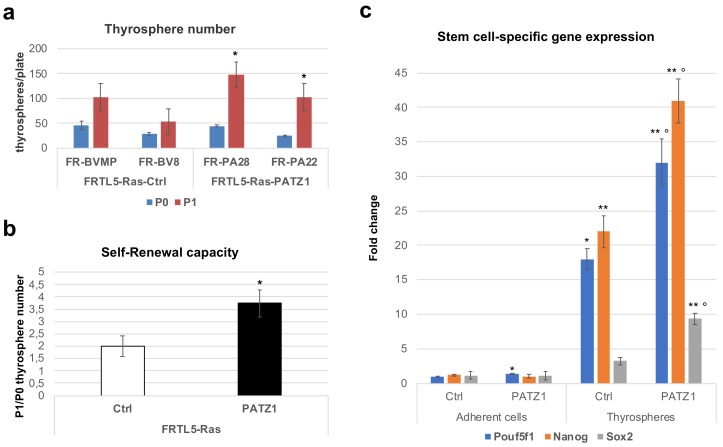
Stemness potential of Ras transformed rat thyroid cells. (**a**) Thyrosphere number was counted in two serial passages every 14 days. P0 = primary thyrospheres; P1 = secondary thyrospheres. Two clones for both FRTL5-Ras-Ctrl (FR-BVMP, FR-BV8) and FRTL5-Ras-PATZ1 (FR-PA28, FR-PA22) cells [19] were analyzed. Mean values ± SE of three independent experiments are reported. *, *P* < 0.05, versus P0 thyrospheres, as assessed by t-test. (**b**) Self-renewal capacity was evaluated by the P0/P1 ratio of thyrospheres number. *, *P* < 0.05; (**c**) qRT-PCR analysis of *Pouf5f*1, *Nanog* and *Sox2* gene expression in P1 thyrospheres and corresponding bulk cells grown in adhesion. The data shown express the mean values ± SE of one of three independent experiments performed in duplicate. The two additional experiments are shown in supplementary Figure S3. *, *P* < 0.05; **, *P* < 0.01 versus related adherent cell controls; *P* < 0.05; *P* < 0.01 *versus* control thyrospheres, as assessed by multiple t-tests.

At 14 days, primary thyrospheres were disaggregated and re-plated (7000 cells/plate) to generate secondary thyrospheres (P1) that were counted at further 14 days from re-plating to calculate their self-renewal capacity, which is defined by the ratio between the number of P1 and P0 thyrospheres. In agreement with a potential role of PATZ1 in the stemness of these cells, FRTL5-Ras-PATZ1 thyrospheres showed an improved capacity to self-renew compared to FRTL5-Ras-Ctrl cells (Figure 3a,b). Consistent with that, the increased expression of stem cell marker genes, such as *Pou5f1*, *Nanog,* and *Sox2*, observed in thyrospheres compared to adherent cells, was enhanced in FRTL5-Ras-PATZ1 secondary thyrospheres compared to their controls (Figure 3c and Figure S3). These results suggest that PATZ1 enhances the stemness potential in FRTL5-Ras cells.

### 3.3. Thyrosphere Diameter is Decreased in PATZ1-Expressing FRTL5-Ras Cells

**Figure 4:**
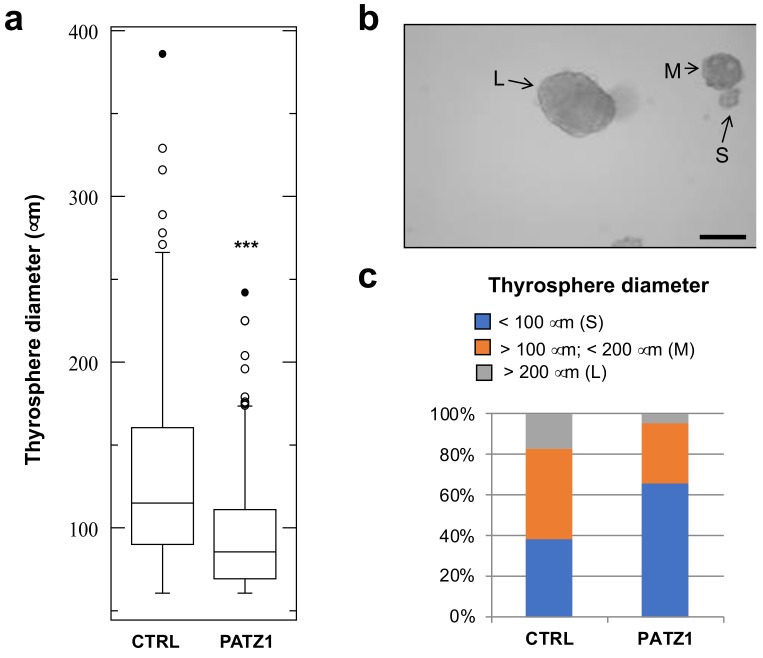
Thyrosphere diameter evaluation. (**a**) Boxplot showing diameter of secondary thyrospheres in two independent clones and two independent experiments for each control and *PATZ1* expressing FRTL5-Ras cell line at 14 days from plating. The graph shows median values ± SD of all spheres with a diameter > 60 μm. ***, *P* < 0.0001. (**b**) Representative image of FRTL-Ras-Ctrl thyrospheres showing different size. L = large sphere (diameter > 200 μm); M = middle sphere (diameter > 100μm < 200 μm); S = small sphere (diameter < 100 μm). Scale bar: 200 μm. (**c**) Distribution of the three thyrosphere subgroups in FRTL5-Ras-Ctrl (CTRL) and FRTL5-Ras-PATZ1 (PATZ1) cells.

Interestingly, the diameter of secondary thyrospheres was not homogenous. As shown in Figure 4a, a significant decrease of sphere diameter was observed in FRTL5-Ras-PATZ1 compared to FRTL5-Ras-Ctrl. We have established a cut-off range for sphere diameter in order to divide thyrospheres in three subgroups: large (diameter > 200 μm); medium (diameter >100 μm < 200 μm); small (diameter < 100 μm) (Figure 4b). Most (63%) of the control spheres were either medium or large, whereas most (71%) of the *PATZ1* expressing spheres were small (Figure 4c). This evidence suggests a reduced proliferation capability of *PATZ1*-expressing cells composing the thyrospheres.

## 4. Discussion

The recent discovery of CSC in a variety of tumors has changed the view of carcinogenesis and therapeutic strategies [25]. According to the CSC theory, a small subpopulation of tumor cells with stem cell like properties can initiate and maintain the whole tumor. Therefore, the identification and characterization of such tumorigenic population represents a crucial step to develop effective therapies [26].

In thyroid cancer, the CSC hypothesis holds that CSC originate either from normal stem cells, progenitor cells, or more mature cells that have dedifferentiated. However, most researchers agree that CSCs, independently of their origin, are the culprits of the cancer [27]. Consistently, a small population of thyroid cells that possess the ability to self-renew and reinitiate serial transplantable tumors that recapitulate the phenotype and metastatic behavior of parental tumors have been isolated in both well-differentiated and anaplastic thyroid carcinomas [28].

FRTL5 rat thyroid cells are an established cell line derived from normal thyroid glands of 5–6-week-old Fischer rats and extensively used to study the molecular mechanisms of neoplastic thyroid transformation. In particular, the stable expression of the Ha-Ras^V12^ oncogene in these cells is able to drive neoplastic transformation towards a malignant undifferentiated phenotype [20,21,22].

Here, we showed for the first time that FRTL5 cells transformed by the *Ha-Ras^V12^* oncogene contain a subpopulation of stem-like cells (~0.5%), which were able to grow as thyrospheres, to increase their number in subsequent passages (indicative of symmetric division), and to enhance the expression of stem cell markers such as *Oct4*, *Nanog*, and *Sox2* compared to the bulk cell line grown in adhesion. To date, only one paper—through the use of FACS followed by mRNA quantification—reported the presence in the FRTL5 cell line of a subpopulation of cells overexpressing stemness genes [29]. Consistently, we isolated thyrospheres from FRTL5 untransformed cells. However, when FRTL5-Ras cells were used, the number and size of thyrospheres were drastically higher with respect to those obtained from parental FRTL5 cells according to their malignant phenotype.

PATZ1 has been shown to be a crucial regulator of embryonic stem cells—being directly involved in the regulation of key pluripotency genes such as *Pou5f1* and *Nanog*—thus suggesting a role in the maintenance of the cell stemness [9]. An important implication of the CSC theory is that CSC are closely related to normal stem cells and likely share many of their features [30]. If this is true, then PATZ1 could be involved in the maintenance of the CSC phenotype. Indeed, we have recently shown that *PATZ1* is overexpressed in the stem cell compartment of human GBM, and its expression correlates with the proneural GBM subtype characterized by a stem cell gene signature [10]. However, survival analysis of GBM patients showed that lower levels of *PATZ1* are associated with the more invasive mesenchymal phenotype, and thus show poor prognosis [10]. Therefore, a dual oncogene/tumor suppressor role has been suggested for *PATZ1* in GBM [6]. A similar role could also be suggested for *PATZ1* in thyroid cancer. Indeed, a tumor suppressor role has been demonstrated for *PATZ1* in vitro and in vivo by using human thyroid cancer, normal cells, and nude mice transplanted with these cells, respectively, where it counteracts EMT and partially induce the reverse process MET [16,17]. Moreover, we recently showed that the loss of one or two alleles of the *Patz1* gene enhances thyroid carcinogenesis in mice transgenic for the *RET/PTC1* oncogene [18]. On the other hand, with the present work, we showed that *PATZ1* enhances the stemness potential of rat thyroid cancer cells and tumor engraftment in nude mice, thus suggesting an oncogenic role. It is known that EMT and MET play important roles in stem cell differentiation and de-differentiation. However, which of them enables cells to obtain the pluripotency still remains controversial, and clarification of the relationship between cellular transformation and pluripotent characters is still a matter of study [31]. Our results are in line with the studies by Ocaña et al. [32], in which repression of EMT reverted the cells to the epithelial phenotype concomitant with the acquisition of the stem cell properties. Nevertheless, the enhanced in vivo engraftment of FRTL5-Ras-PATZ1 compared to FRTL5-Ras-Ctrl cells is not coupled with an enhanced tumor growth. Indeed, the median tumor growth is significantly lower in FRTL5-Ras-PATZ1 than in FRTL5-Ras-Ctrl mice, likely due to a 50% of mice in which the tumor growth was extremely low or regressed up to complete disappearance. Moreover, all three tumors derived from FRTL5-Ras-Ctrl cells showed a positive immunostaining for the proliferation marker Ki67, while only one out of three tumors derived from FRTL5-Ras-PATZ1 cells was Ki67-positive, and none of FRTL5-Ras-PATZ1 xenografts showed increased apoptosis compared to controls. These data, even though limited by the low number of tumors that we could analyze ex-vivo, support an inhibitory role of PATZ1 on tumor cell proliferation and are consistent with our previous results showing that *PATZ1* expression inhibits in vitro cell proliferation of FRTL5-Ras cells [19]. Furthermore, as consistent with previous studies [16,17,18], *PATZ1* expression tends to be lost in vivo. It is likely downregulated as a consequence of the tumor aptitude to select cells that have a higher proliferative potential.

Noteworthily, despite the enhanced stemness potential of *PATZ1*-expressing cells—testified by the enhanced self-renewal capacity and stem cells markers expression of the thyrospheres compared to controls—the size of *PATZ1*-expressing thyrospheres was significantly smaller than control spheres, which suggests an inhibition of their proliferation. Notably, one of the FRTL5-Ras-PATZ1 tumors showed signs of MET. A larger number of animals should be analyzed in a further study to validate a potential role of PATZ1 in the acquisition of this phenotype. However, a similar result was previously shown in xenograft tumors derived from PATZ1-expressing human ATC cells, where a key role of PATZ1 in counteracting the EMT has been demonstrated [16,33].

In conclusion, we demonstrated that PATZ1 expression in FRTL5-Ras cells enhances stemness potential and, as a likely consequence of that, tumor engraftment in nude mice. However, the presence of PATZ1 inhibits sphere growth in vitro and tumor growth in vivo. Therefore, we hypothesize that the dual oncogene/tumor suppressor role of PATZ1 is exerted in two different cell populations within the tumor: the oncogenic role, on the CSC, contributes to the maintenance of the stemness, thus enhancing the in vivo engraftment of the tumor; the tumor suppressor role, on bulk cancer cells, inhibits proliferation and reverts the transformed phenotype. Since only few *PATZ1* mutations have been so far reported in thyroid cancer (5 out of 50 PTCs, according to the International Cancer Genome Consortium-ICGC data portal [34]), our idea is that PATZ1 is mostly epigenetically regulated during cancer progression. Accordingly, we previously showed a Ras-miR-29b axis involved in PATZ1 downregulation during thyroid cancer cell transformation, but it is likely that further mechanisms are involved. Future experiments are needed to validate such hypothesis and investigate in further details the duplicity of PATZ1 functions, as well as the switch from high to low PATZ1 expression in the context of cancer progression.

## Supplementary Materials

Supplementary materials can be found at http://www.mdpi.com/2073-4425/10/2/127/s1.
