# Supplementary material for: Dual Oncogenic/Anti-Oncogenic Role of PATZ1 in FRTL5 Rat Thyroid Cells Transformed by the Ha-RasV12 Oncogene
Source: Genes (Basel). 2019 Feb 9;10(2):127. doi: 10.3390/genes10020127 (PMC6410289; doi:10.3390/genes10020127)
Supplement: Supplementary file 1 [file genes-10-00127-s001.zip › supplementary Figures/Figure S1.pdf]

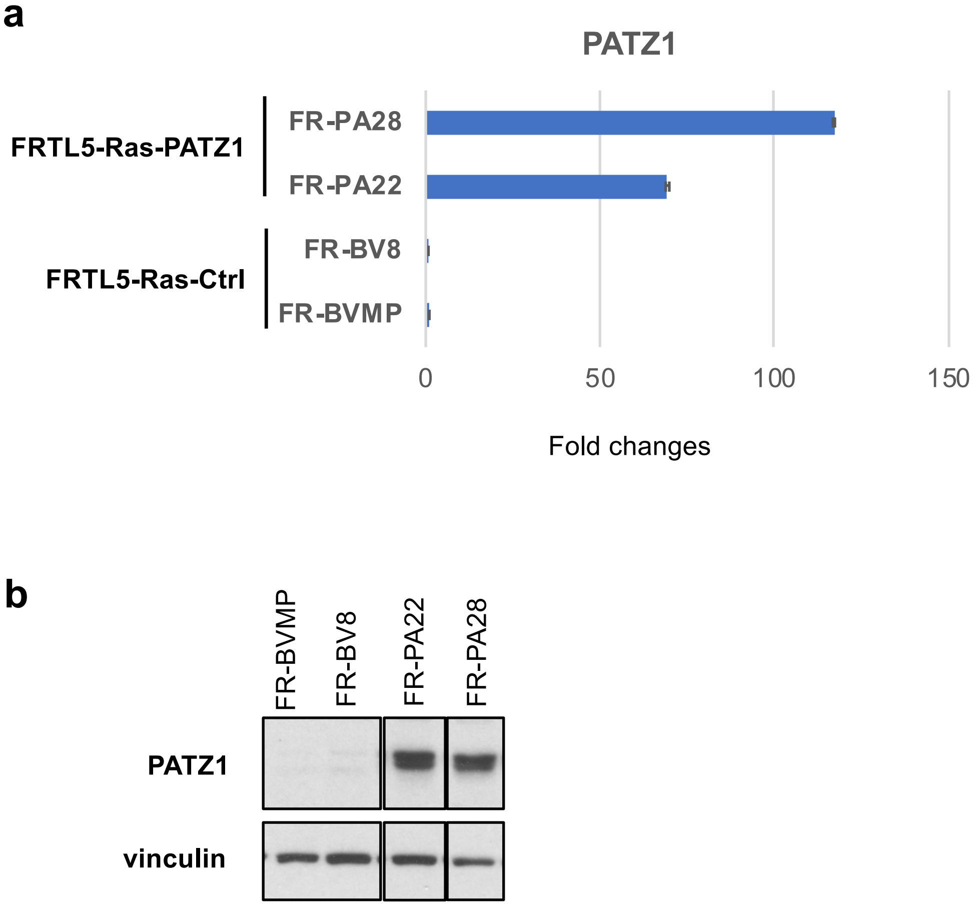

Figure S1. PATZ1 expression in FRTL5-Ras-PATZ1 cells. (a) qRT-PCR and (b) western blot analysis of PATZ1 expression in FRTL5-Ras-Ctrl and FRTL5-Ras PATZ1 clones used in this study [19]. *G6pd* and Vinculin have been analyzed as sample loading controls in a and b, respectively. Black lines delineate the boundary between not contiguous lanes of the same gel.
