# Supplementary material for: Dual Oncogenic/Anti-Oncogenic Role of PATZ1 in FRTL5 Rat Thyroid Cells Transformed by the Ha-RasV12 Oncogene
Source: Genes (Basel). 2019 Feb 9;10(2):127. doi: 10.3390/genes10020127 (PMC6410289; doi:10.3390/genes10020127)
Supplement: Supplementary file 1 [file genes-10-00127-s001.zip › supplementary Figures/Figure S2.pdf]

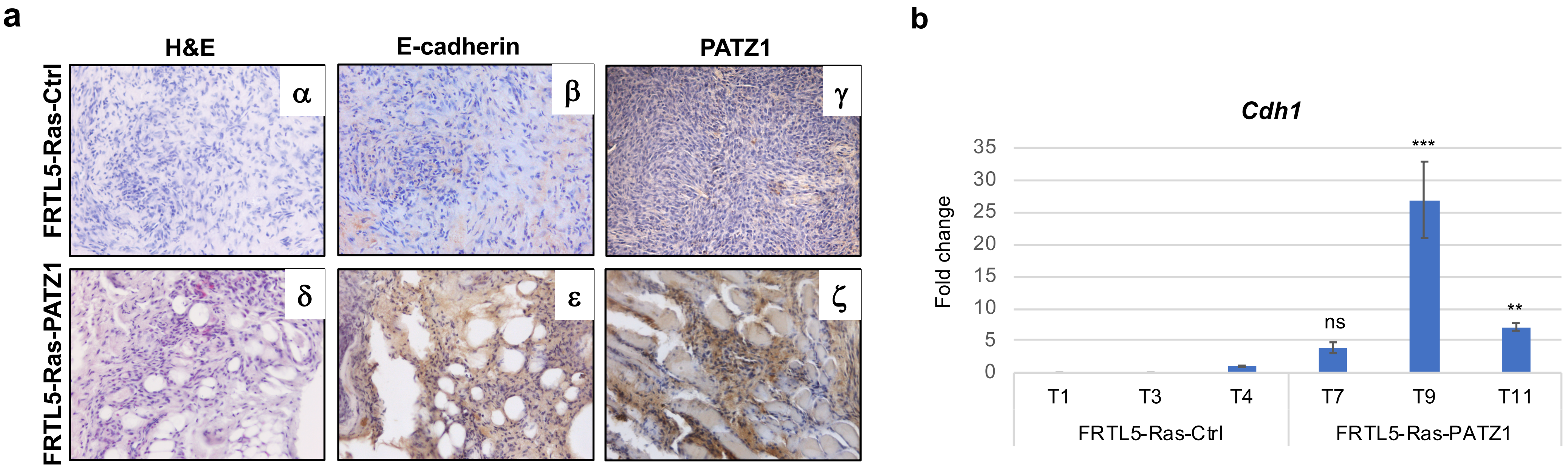

Figure S2. Tumor xenograft phenotype. (**a**) Representative images of Hematoxylin & Eosin staining (H&E), and E-cadherin and PATZ1 immunostaining of tumor xenografts from control (α, β, γ) and PATZ1-expressing (δ, ε, ζ) FRTL5-Ras cells. Note the different architecture resembling follicular-like structures, which stain positively for both E-cadherin and PATZ1, in the FRTL5-Ras-PATZ1 specimens. Original magnification: 20x. (**b**) qRT-PCR analysis of *Cdh1* (E-cadherin) gene expression in tumor xenografts from FRTL5-Ras-Ctrl and FRTL5-Ras-PATZ1 cells. The data shown express the mean values +/- SE of three or four independent experiments. Note that in two out of three tumors from the control group (T1, T3) the *Cdh1* expression was undetectable. Statistical differences were assessed with the respect to T4 through one-way ANOVA followed by Tukey's multiple comparison test. ns, not significant; ***, $P < 0.001$; **, $P < 0.01$
