# Supplementary material for: Dual Oncogenic/Anti-Oncogenic Role of PATZ1 in FRTL5 Rat Thyroid Cells Transformed by the Ha-RasV12 Oncogene
Source: Genes (Basel). 2019 Feb 9;10(2):127. doi: 10.3390/genes10020127 (PMC6410289; doi:10.3390/genes10020127)
Supplement: Supplementary file 1 [file genes-10-00127-s001.zip › supplementary Figures/Figure S3.pdf]

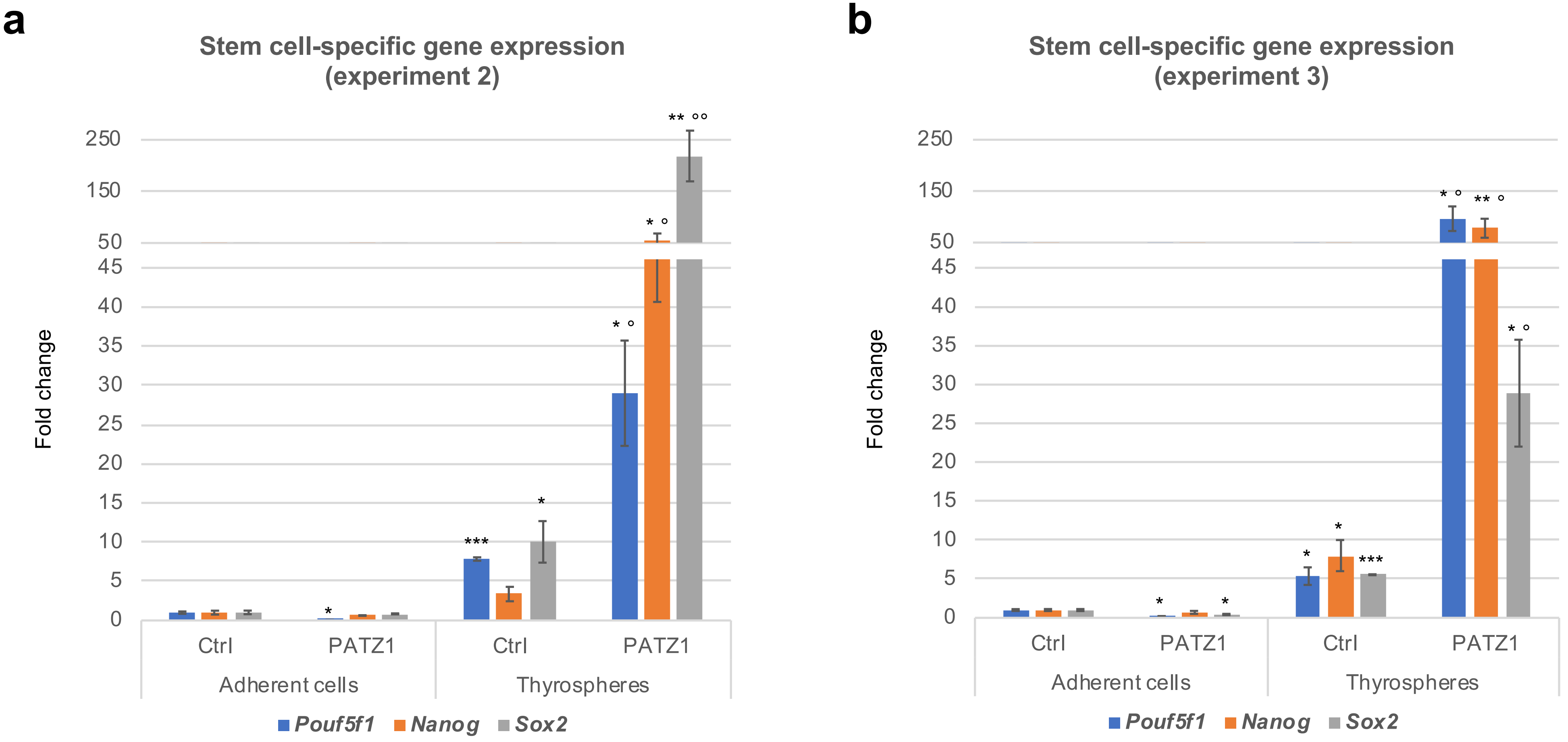

Figure S3. Stem-cell markers expression in adherent and spheroid FRTL5-Ras-PATZ1 cells**.** Two out of three independent experiments of qRT-PCR to analyze *Pouf5f1*, *Nanog* and *Sox2* gene expression in P1 thyrospheres and corresponding bulk cells grown in adhesion. Each panel shows mean values +/- SE of one experiment performed in duplicate. *, P < 0.05; **, P < 0.01 *versus* related adherent cell controls; °, P < 0.05; °°; P < 0.01 *versus* control thyrospheres, as assessed by multiple t-tests. See also Figure 3c for another replica experiment.
